# Recommendations on the use of the flash continuous glucose monitoring system in hospitalized patients with diabetes in Latin America

**DOI:** 10.1186/s13098-024-01362-4

**Published:** 2024-06-12

**Authors:** Ana María Gómez, Diana Cristina Henao Carrillo, Matías Alberto Ré, Raquel N. Faradji, Oscar Flores Caloca, Natalia Eloísa de la Garza Hernández, Carlos Antillón Ferreira, Juan C. Garnica-Cuéllar, Marcio Krakauer, Rodolfo J. Galindo

**Affiliations:** 1 Pontificia Universidad Javeriana, Bogotá, Colombia; 2 Hospital San Juan De Dios La Plata, La Plata, Argentina; 3 Clínica EnDi, Centro Médico ABC, CDMX, Mexico; 4 TEC Salud Monterrey NL, Monterrey, Mexico; 5 CEMEDIN, Monterrey, Mexico; 6 Centro Médico ABC Santa Fe, CDMX, Mexico; 7 Centro Médico Nacional 20 de Noviembre, ISSSTE, CDMX, Mexico; 8 Sociedade Brasileira de Diabetes, São Paulo, Brazil; 9 University of Miami Miller School of Medicine, Miami, USA

**Keywords:** Diabetes, Flash continuous glucose monitoring system, Glucose monitoring, Hospitalization

## Abstract

**Background:**

Continuous glucose monitoring can improve glycemic control for hospitalized patients with diabetes, according to current evidence. However, there is a lack of consensus-established recommendations for the management of hospitalized patients with diabetes using flash continuous glucose monitoring system (fCGM) in Latin America. Therefore, this expert consensus exercise aimed to establish guidelines on the implementation of fCGM in the management of hospitalized patients with diabetes in Latin America.

**Methods:**

The modified Delphi method was applied on a panel of nine specialists, establishing consensus at 80%. A twenty-two-question instrument was developed to establish recommendations on the use of fCGM in hospitalized patients living with diabetes.

**Results:**

Based on consensus, experts recommend the use of fCGM in hospitalized patients with diabetes starting at admission or whenever hyperglycemia (> 180 mg/dl) is confirmed and continue monitoring throughout the entire hospital stay. The recommended frequency of fCGM scans varies depending on the patient's age and diabetes type: ten scans per day for pediatric patients with type 1 and 2 diabetes, adult patients with type 1 diabetes and pregnant patients, and seven scans for adult patients with type 2 diabetes. Different hospital services can benefit from fCGM, including the emergency room, internal medicine departments, intensive care units, surgery rooms, and surgery wards.

**Conclusions:**

The use of fCGM is recommended for patients with diabetes starting at the time of admission in hospitals in Latin America, whenever the necessary resources (devices, education, personnel) are available.

## Introduction

Patients diagnosed with diabetes have a higher risk of complications and hospitalizations [1]. Hospital length-of-stay in patients with diabetes are longer than those without diabetes and more likely to be admitted to the intensive care unit (ICU) [2]. Furthermore, patients with hyperglycemia have an associated increased mortality risk [3].

Continuous glucose monitoring (CGM) provides frequent measurements of interstitial glucose levels, as well as information on the direction and magnitude of glucose trends. The use of CGM has demonstrated to decrease blood glucose excursions, lower HbA1c values, and reduce hypoglycemic episodes, which together diminish the risk of complications associated with diabetes. In addition, use of CGM helps in reducing glucose variability [4–7].

In hospital settings, the integration of a CGM system into a glucose telemetry system has demonstrated a reduction on the risk of inpatient hypoglycemia, particularly recurrent hypoglycemic events [8, 9].

There are two basic types of CGM devices. The first type includes those that are owned by the user, unblinded, and intended for frequent or continuous use, including real-time CGM (rtCGM) and intermittently scanned CGM (isCGM). The second type is professional CGM devices that are owned by practices and applied in the clinic, which provide data that are blinded or unblinded for a discrete period of time. The types of sensors currently available are either disposable (rtCGM and isCGM) or implantable (rtCGM). One specific isCGM device (Freestyle Libre 2) and three specific rtCGM devices (Dexcom G6, Dexcom G7, and FreeStyle Libre 3) have been designated integrated CGM devices [8].

During the COVID-19 pandemic, many healthcare institutions incorporated CGM to manage diabetes in patients, reducing the burden of inpatient care and minimizing direct contact between healthcare professionals and patients [10].

However, clinical evidence and recommendations on the management of glycemia with flash glucose monitoring (fCGM) in hospitalized patients living with diabetes is scarce.

The objective of the present work is to provide recommendations on therapeutic goals and the management of patients with diabetes using the fCGM on a hospital setting.

## Methods

A systematic review of Clinical Practice Guidelines (CPG) and consensus guidelines for hospitalized patients with diabetes was conducted. This review involved an exhaustive search of various medical databases, including PubMed, Cochrane Library, and EMBASE, to identify relevant guidelines published in the last ten years. The search terms included “diabetes,” “hospitalized patients,” “clinical practice guidelines,” and “consensus guidelines.” Inclusion criteria were established to select guidelines specifically addressing the management of diabetes in a hospital setting [1, 4, 8, 11].

Following the systematic review, a multidisciplinary committee was formed to develop a checklist of relevant aspects derived from the identified guidelines. The committee included endocrinologists, diabetologists and internal medicine physicians. The formation and composition of the committee ensured a comprehensive perspective on the essential elements of diabetes care in hospitalized patients.

Afterwards, the modified Delphi panel method was applied to obtain consensus and recommendations related to glucose monitoring and follow-up of hospitalized patients with diabetes. The Delphi method is a prospective research alternative to obtain a reliable consensus on expert opinions. After rounds of discussions, the committee arrived at final statements and a rating was assigned by every member to each statement [1, 6, 12, 13].

The consensus meeting was held with a group of nine experts, including endocrinologists, pediatric endocrinologists and internal medicine physicians, with expertise in management of pediatric and adult patients with diabetes in a hospital setting, with public and/or private inter-institutional experience.

The instrument consisted of 22 questions evaluating perception through numeric scales and classifying their agreement with statements related to management goals and use of the fCGM in a hospital setting. This final aspect was assessed for pediatric, adolescent, and adult patients with Type 1 diabetes mellitus (T1DM) and Type 2 diabetes mellitus (T2DM) in a hospital setting.

A priori consensus was established at 80%, considering that the Delphi panel was performed during an in-person session, once the first section of the instrument was covered, a quality-control analysis of the data was performed followed by descriptive statistical analysis using central tendency and dispersion metrics and stack classification to identify perception tendencies of the attributes evaluated semiquantitative and qualitatively in free lists.

## Results

Based on the consensus building exercise, we stablish the following recommendations of fCGM in the management of hospitalized patients with diabetes in Latin America.

- *Glycemic targets for in-hospital management*

Recommendations:

1. Up to 86% of panelists agreed with ADA criteria on glycemic control objectives [8].
2. In hospitalized pediatric patients with T1DM and T2DM, and adults with T1DM; the recommendation was for an average of seven evaluations of point-of-care capillary glucose (POC BG) a day, and 4 POC BG for adults with T2DM. For pregnant patients with T2DM, eight evaluations POC BG should be the average and the minimum for those living with T1DM **(Consensus: 100%).**

b. *Management goals*

fCGM allows for frequent glucose monitoring that allow the evaluation on the effects of treatment modifications, diet and exercise have on glucose levels. Also, is especially important to monitor for and prevent hypoglycemia and hyperglycemia in patients with diabetes [8, 11, 14].

Recommendations

1. It is recommended that the target glucose range for both critically ill and noncritically ill patients should be between 140–180 mg/dl. For some patients, more strict goals of 110–140 mg/dl may be necessary, as long as it can be achieved without causing significant hypoglycemia. (Consensus:100% consensus).

c. *Candidates for glycemic control through fCGM*

Patient characteristics and scenarios where the use of fCGM should be recommended were discussed by diabetes type and age group, clinical benefits where the panel reached consensus are listed in Table 1 [8].

**Table 1:** Clinical benefits on the use of fCGM

Population	Benefits	Consensus
All patients with diabetes	Improve time in range Reduce acute complications fCGM as an education tool Lifestyle changes Improve condition understanding Improve treatment adherence Aid glycemic control improvement Decreases hospitalization duration Reduce risk of adverse events related to hypo or hyperglycemia 24 h glucose level monitoring without patient disturbance Patients and clinicians can check glucose levels between capillary glucose checks Reduce number of capillary glucose evaluations Patients and clinicians can check glucose trends and take early measures Patient empowerment for self-management Ease of application and low risk Reduce maternal–fetal complications during pregnancy	High (100%)
Pediatrics and teenagers with T1DM or T2DM	Improved metabolic and glycemic control Reduce risk of hypoglycemia Patient education for decision-making processes Reduce number of hospitalizations Patient empowerment	High (100%)
Adults with T1DM	Improved metabolic and glycemic control Reduce risk of hypo and hyperglycemia Improve quality of life Decrease needs for nursing personnel monitoring during hospitalizations	High (100%)
Adults with T2DM	Improved metabolic and glycemic control Reduce risk of hypo and hyperglycemia Improve quality of life Decrease needs for nursing personnel monitoring during hospitalizations	High (100%)

### fCGM inpatient management recommendations

#### Patients with T1DM

1. The choice of fCGM device should be tailored to the individual patient’s needs, preferences, and clinical conditions. However, it is equally important to consider the healthcare staff's familiarity with the device, the ease of use, and the level of training required to effectively manage and interpret fCGM data (100% consensus).
2. Device selection should be through a shared decision-making process to identify the most appropriate device (100% consensus).

#### Patients with T2DM

1. fCGM should be recommended in patients managed with basal bolus insulin regimen, recurrent or severe hypoglycemia, and those who have a condition or disability (including learning disabilities or cognitive impairment) where self-management and glucose monitoring cannot be performed by themselves through capillary glucose evaluations (100% consensus).

d. *Considerations for glycemic management using fCGM in the hospital*

The panel discussed all hospital settings and patient types where fCGM recommendations should be considered and special considerations when evaluating fCGM use. All scenarios where the panel reached 100% consensus were included (Table 2).

**Table 2:** Candidate considerations in hospital settings and considerations prior to utilization (Consensus:100%)

Candidate considerations for fCGM in hospital settings	Special considerations prior to utilization
Patients managed through fCGM: continue current monitoring during hospitalizations	Requirements: Evaluate device requirements, such as access to specific technology (smartphones or specific software)
Third-party notifications: cases where a caregiver requires notification of alerts or predictive alarms provided by the device	Data collection method: device compatibility with other technologies and whether data can be shared with medical care providers to inform treatment adjustments Education and training: patients and caregivers need to receive initial and ongoing education and training to monitor and adjust therapy. Training on alarm/alert settings when initiating CGM is crucial to avoid alarm overload
Patient on a hybrid closed loop system: treatment should continue as-is unless contraindicated	Patient lifestyle: Unpredictability of patient’s activities and serum glucose, variability effects in quality of life
Pregnancy and diabetes: time in range (63–140 mg/dl) goal is above 70%	Equipment maintenance: evaluate compatibility of the frequency of sensor replacement and patient’s lifestyle and means
Elective surgery: in patients with HbA1c is outside of goal range	Atopy or sensitivity: account for potential allergies and skin reactions (e.g. local skin reactions)
Stress hyperglycemia: consider fCGM during hospitalizations	Lesions: fCGM devices should not be inserted into an area of generalized edema or cellulitis
	Procedures: In patients scheduled for a procedure or surgery, the sensor must be placed on a different body area (contralateral side) from where the procedure will take place

e. *fCGM management in hospital setting*

### fCGM in hospital settings recommendations

1. When possible, fCGM should be performed alongside traditional capillary glucose monitoring (100% consensus).
2. Nursing staff should be in charge of data collection of hospitalized patients (patient identification information, vital signs, glycemic data, clinical status. medications), preferably in a registration sheet at least every 8 h (100% consensus).
3. In pediatric patients with T1DM and T2DM, a minimum of nine fCGM scans a day are recommended, ten for adults with T1DM, seven for adults with T2DM and ten for pregnant patients (100% consensus).
4. Independent of glycemic control, fCGM should start at the time of admission and during the entire hospital stay if resources are available. Other aspects to consider are: reason for hospitalization and complications. Data to be collected and interpreted is listed in Table 3 (100% consensus).
5. fCGM data should be evaluated by the medical team at least every 12 h, with more frequent evaluations as needed based on the patient's clinical status. Insulin dose adjustments must be performed accordingly to ensure optimal glycemic control (100% consensus).
6. Report analysis and treatment adjustment should be dynamic, with daily evaluations and record history development to provide datapoints for analysis of identified diabetes patients (100% consensus).
7. Scenarios to recommend fCGM in a hospital setting include patients with CGM prior to hospitalization, uncontrolled patients, those requiring therapeutic adjustments or presenting with hyper and hypoglycemia (100% consensus).
8. Hospital settings that could benefit from the use of fCGM include the emergency room, internal medicine, intensive care, maternity ward, surgery rooms and the surgery ward, especially in cases of elective surgery and non-critical care where it could improve detection of hypo and hyperglycemia episodes and hypoglycemia prevention (100% consensus).
9. Once the patient is discharged, if resources are available, the sensor can remain in place. Follow-up should be scheduled within 10 days to evaluate the ambulatory glucose profile or according to the sensor's life span, which is typically 14 days. (100% consensus).

**Table 3:** Data to evaluate through fCGM in hospitalized patients

Data	Consensus
Time of use of fCGM over 70% based on days of sensor availability	High (89%)
Glycemic variability determined by variation coefficient and standard deviation of average value	High (89%)
Percentage of time in range (> 70% of the time)	High (89%)
Percentage of time below range (< 4% of the time)	High (89%)
Percentage of time above range (< 25% of the time)	High (89%)
AGP evaluation for hypoglycemia detection	High (100%)
AGP evaluation for hyperglycemia detection	High (100%)
AGP evaluation for glycemic variability identification	High (100%)
Comparison with prior data for change evaluation	High (89%)

Additionally, glycemic control objectives recommendations were developed through the discussion and evaluation of published guidelines for hospitalized patients with diabetes. The recommendations where the panel reached consensus are displayed on Table 4.

**Table 4:** Hospitalization setting glycemic control goals

Recommendation	Consensus
The patient must provide authorization for their data to be shared with hospital personnel (if not previously provided)	High (89%)
For non-critical patients with diabetes, we recommend intensive or standard glycemic control based on resource availability, glucose level evaluation, patient status, and acceptability	High (89%)
Glycemic level goals should be 100 to 180 mg/dl in acute or severe conditions	High (89%)
In patients with acute conditions, a glycemic range of 70–100 mg/dL and a downward trend indicate imminent hypoglycemia. In such cases, a clinician should evaluate the patient, and the team should discuss management	High (89%)
As clinicians gain experience with fCGM, these could be linked to the patient’s electronic clinical records	High (89%)
Due to the risk of inaccuracy during acute conditions, capillary blood glucose should be analyzed at least twice a day in patients with fCGM	High (89%)
During admission, patients should receive information on the need for capillary blood glucose monitoring during hospitalization for safety reasons and to alert personnel of results outside of the goal range	High (89%)
Nursing personnel should be aware of the need for additional capillary blood glucose evaluations when there is a discrepancy between readings and the patient’s symptoms	High (89%)
In cases where electronic documentation is not available, it is recommended to obtain at least three fCGM readings: fasting, pre-meal, and bedtime	High (89%)
Unless incapacitated or gravely ill, most patients using fCGM can continue using it during hospitalization	High (89%)
When selecting a fCGM, we recommend a shared decision-making process to identify the patient’s needs and preferences and provide an appropriate device recommendation	High (89%)

POC BG is recommended to confirm before continuation of fCGM use in special scenarios (e.g., severe hypotension, after surgery, cardiac arrest, etc.); to confirm hypoglycemia and monitor recovery; as well as cases where symptoms do not match sensor glucose report or the reading seems unlikely in the circumstances (e.g., if symptoms of hypoglycemia are present but the sensor glucose reading is normal); if the sensor reading is unreliable or obviously erroneous (e.g., sensor does not display reading, or the trend arrow is absent) and during and after exercise [6].

It is possible to notice a difference between fCGM and POC BG. However, we consider a difference acceptable if it is within ± 20% of the absolute difference between fCGM and POC BG that are greater than 100 mg/dL, or within ± 20 mg/dL of the absolute difference between fCGM and POC BG if capillary blood glucosa is equal to or less than 100 mg/dL. This definition is based on the reference standard for integrated CGM devices, and it is known as %20/20 [15].

## Discussion

The expert panel recommended the use of fCGM in hospital settings in patients with T1DM or T2DM, and, in pregnant women with a number of scans that ranges from 7 to 10 depending on patient type. Ambulatory use of fCGM systems have demonstrated improvements in glucose management and patient satisfaction, reduced fear of hypoglycemia, improved quality of life, and reduction of diabetic emergency hospitalizations [14].

Traditional glucose evaluations can overlook hyper and hypoglycemia during admission specially when asymptomatic [1]. A retrospective study on adult patients with T2DM compared the use of fCGM to capillary glucose monitoring. Outcomes were changes in acute diabetes-related events and all-cause inpatient hospitalizations, occurring during the first 6 months after acquiring the fCGM compared with event rates during the 6 months prior to system acquisition. Acquisition of the flash CGM system was associated with reductions in acute diabetes-related events and all-cause inpatient hospitalizations. Acute diabetes-related events rates decreased from 0.180 to 0.072 events/patient-year (hazard ratio [HR]: 0.39 [0.30, 0.51]; *P* < 0.001) and all-cause inpatient hospitalizations rates decreased from 0.420 to 0.283 events/patient-year (HR: 0.68 [0.59 0.78]; *P* < 0.001) [14].

In a pilot study using fCGM in hospitalized patients with COVID-19, a high rate of acceptance among patients was reported (80%). Percentage of time in hyperglycaemia exhibited statistically significant associations with both percentage of time in hypoglycaemia (p = 0.035) and percentage of time in range (p = 0.005), as well as with HbA1c (p = 0.004) and average glucose (p < 0.0001). Finally, the average glucose was also significantly associated with percentage of time in hypoglycaemia (p = 0.003), percentage of time in range (p = 0.01), and HbA1c (p = 0.046). These providing an innovative approach for hospitalized patients with diabetes in different scenarios where glucose control remains a key element of their management [16].

We recognized some limitations, including the lack of participation of nursing staff and diabetes educators. We, however, included several experts from different hospital settings and institutions, both public and private, with experience in the management of hospitalized patients with diabetes.

Although recommendations have been published, in Latin America there are no specific guidelines for hospitalized patients with diabetes in which glycemic control objectives are evaluated. This highlights the need to update and reach consensus on some parameters that are useful and practical as clinical outcomes in inpatients. Therefore, after careful consideration of available evidence and considering the Latin American context through the incorporation of expert’s opinion, the present consensus recommends the use of fCGM in hospitalized patients with diabetes, as it allows more detailed glucose assessment and has the potential of reducing the length of hospital stays.

## Conclusions

The expert panel recommends the use of fCGM for patients with diabetes starting at the time of admission and during the entire hospital in Latin America, whenever the necessary resources (devices, education, personnel) are available.

Expanding the use of fCGM could have the additional benefit of contributing valuable insight into glycemic parameters dynamics in different clinical scenarios within Latin American and improving diabetic care. There are new generations of fCGM that have additional features like alarms and real time transmission of glucose which could improve the management of these patients.

## Data Availability

Not applicable.

## Abbreviations

ADA: American Diabetes Association
AGP: Ambulatory glucose profile
CPG: Clinical practice guidelines
CGM: Continuous glucose monitoring
A1c: Glycated hemoglobin A1c
GV: Glycemic variability
fCGM: Intermittent scanning continuous glucose monitoring or flash continuous glucose monitoring
JBDS-IP: Joint British Diabetes Societies for Inpatient Care
RAND: Research and Development Center
SMBG: Self-monitoring blood glucose
TAR: Time above range
TBR: Time below range
TIR: Time in range
T1DM: Type I diabetes
T2DM: Type 2 diabetes
UCLA: University of California in Los Angeles
